# Advancements in the study of inward rectifying potassium channels on vascular cells

**DOI:** 10.1080/19336950.2023.2237303

**Published:** 2023-07-18

**Authors:** Chunshu Li, Yan Yang

**Affiliations:** Key Lab of Medical Electrophysiology of Ministry of Education and Medical Electrophysiological Key Lab of Sichuan Province, Collaborative Innovation Center for Prevention and Treatment of Cardiovascular Disease, Institute of Cardiovascular Research, Southwest Medical University, Luzhou, China

**Keywords:** Inward rectifier potassium channels (Kir channels), vascular endothelial cells, vascular smooth muscle cells, vascular stem cells

## Abstract

Inward rectifier potassium channels (Kir channels) exist in a variety of cells and are involved in maintaining resting membrane potential and signal transduction in most cells, as well as connecting metabolism and membrane excitability of body cells. It is closely related to normal physiological functions of body and the occurrence and development of some diseases. Although the functional expression of Kir channels and their role in disease have been studied, they have not been fully elucidated. In this paper, the functional expression of Kir channels in vascular endothelial cells and smooth muscle cells and their changes in disease states were reviewed, especially the recent research progress of Kir channels in stem cells was introduced, in order to have a deeper understanding of Kir channels in vascular tissues and provide new ideas and directions for the treatment of related ion channel diseases.

## GRAPHICAL ABSTRACT

**Figure:**
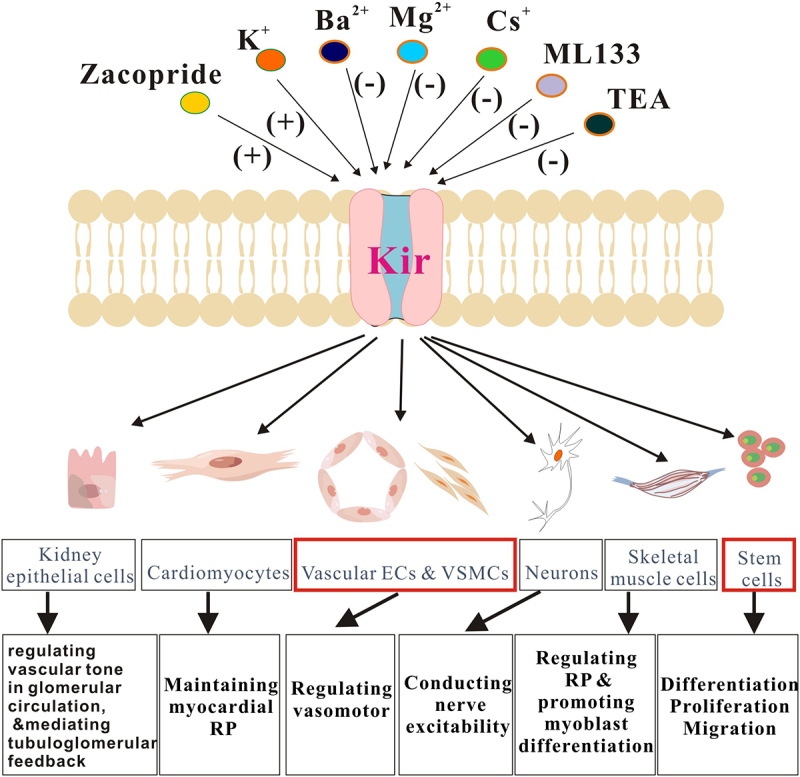

## Introduction

Potassium ion channels (K^+^ channels) play a crucial role in cellular signaling and are essential for maintaining normal cellular function. As research on K^+^ channels continues to advance, an increasing number of ion channels have been discovered and thoroughly studied. Inward rectifier potassium channels (Kir channels) are a type of K^+^ channels that play a critical role in electrical signaling in cells. They are ion channels that allow the selective passage of K^+^ across the plasma membrane of a cell. These channels are referred to as “inward rectifiers” because they are highly selective for inward K^+^ currents, meaning they allow the flow of K^+^ ions into the cell but not out of the cell. These channels are responsible for maintaining the stability of the excitation and resting membrane potential (RP) of excitable tissue cells within living organisms, and are critical for the regulation in homeostasis, and signal transduction [1]. There are currently 15 subtypes of Kir channels that have been discovered. These subtypes are classified into 7 subfamilies based on their function and properties. Additionally, these subfamilies are further grouped into 4 functional categories, which include classic Kir channels, G-protein-gated K^+^ channels, ATP-sensitive K^+^ channels, and K^+^ transport channels [2]. The distribution and phenotype of Kir channel subtypes vary across different cells, resulting in distinct biological functions of Kir channels. Recent studies have focused on discovering new functions and understanding the functional regulatory mechanisms of Kir channels. In this review, we explore the fundamental structure, physiological function, and pharmacological traits of Kir channels. Moreover, we extensively explore the roles of Kir channels in both vascular smooth muscle cells (VSMCs) and vascular endothelial cells (ECs) and their implications in the occurrence and development of various diseases. Additionally, a comprehensive overview of the current research progress on Kir channels in stem cells is provided.

## Basic structure, physiological function and pharmacological characteristics of Kir channels

Kir channels play an essential role in regulating various physiological processes, such as neuronal excitability, cardiac function, and insulin secretion, and are a critically important group of K^+^ channels. The structure of Kir channels consists of four subunits arranged in a tetrameric configuration around a central pore. Each subunit comprises two transmembrane domains connected by a pore-forming loop region. The transmembrane domain is composed of outer (TM1) and inner (TM2) membrane spanning helices, with two short additional helical elements, the slide and the pore helices. Kir channels are distinguished from other potassium channels by their biophysical property of inward rectification that allows more potassium ions to enter into the cell at the membrane potential (Vm) below the equilibrium potential of K^+^ (E_K_) than to leave the cell at Vm above E_K_. Kir channel structures lack the S4 voltage sensor region. As a result, Kir channels are insensitive to membrane voltage and would be active at all Vm. Additionally, Kir channels have two gating mechanisms, the slow gating that opens at long intervals and the fast gating that opens in bursts [1,3]. The regulation of Kir channels involves both the interior and exterior of the transmembrane domain, where the hydrogen bond interconnecting these domains plays a crucial role in gating all Kir channels. The TM1-TM2 hydrogen bond (H-bonding) stabilizes the closed state of the channel and its rupture may be one of the rate-limiting steps in channel transition between the closed to the open conformations. Study showed that this proposed H-bonding interaction determines Kir channel pH sensitivity, pH and PIP2 gating kinetics, as well as a K^+^-dependent inactivation process at the selectivity filter and therefore many of the key regulatory mechanisms of Kir channel physiology [4]. In addition, the Kir channels feature a columnar structure in the cytoplasmic domain that is formed by the combination of the carboxyl and amino termini of each subunit around the cytoplasmic pore, thus enhancing the domain’s stability.

Physiologically, Kir channels play a crucial role in regulating many cellular processes. They are responsible for maintaining RP of cells, modulating the duration and amplitude of action potential, and regulating synaptic transmission. Additionally, the Kir channel is pivotal in maintaining heart rate and rhythm, as well as controlling insulin secretion by pancreatic beta cells. Moreover, the significance of Kir channels in vascular tissue cannot be overstated and will be discussed below. The different subtypes of Kir channels exhibit unique properties, distribution patterns, and functions. In cardiac muscle cells, Kir2.1, Kir2.2, and Kir2.3 are present and play a crucial role in maintaining RP [5]. Vascular ECs and VSMCs express a variety of Kir channels, which participate in the regulation of vasomotor [6]. In neurons, Kir2.1 is lowly expressed, while Kir2.2, Kir2.3, and Kir2.4 are abundant and involved in nerve excitability conduction [7]. The Kir2.x channels are found in skeletal muscle cells and are responsible for regulating RP and promoting myoblasts’ differentiation [8]. The epithelial cells of the kidney rely on Kir channels participate in the regulation of vascular tone in the glomerular circulation, and they are involved in the mechanisms mediating tubuloglomerular feedback, providing K^+^ to the Na^+^-K^+^-2Cl^−^ cotransporter and generate a lumen-positive transepithelial voltage [9].

Pharmacologically, multiple substances play a crucial role in regulating the activity of Kir channels. Both the transmembrane and cytoplasmic regions of Kir channels are affected by Mg^2+^ and polyamines, which physically prevent K^+^ penetration [10]. Additionally, an increase in extracellular K^+^ concentration can enhance the activation of Kir channels. Kir channels are responsive to Ba^2+^ and Cs^+^ and can be effectively blocked by these elements. However, it is worth noting that different subtypes of Kir channels exhibit varying degrees of sensitivity to these inhibitors, with the disparity being attributed to the heterogeneous assembly of kir2.x subunits [1]. Studies have also shown that Mepyramine and Diphenhydramine can both block kir2.x channels, although their action is not subtype-specific. Chloroquine, on the other hand, is known to interact with polyamine binding sites in the cytoplasmic region to block kir2.1 channels and potentially cause arrhythmia [11]. Furthermore, *N*-[(4-Methoxyphenyl) methyl]-1-naphthalenemethanamine Hydrochloride (ML133) has been found to selectively inhibit kir2.x channels and depolarize the membrane in a dose-dependent manner. The proper activation of Kir channels is dependent on the presence of phosphatidylinositol 4, 5-biphosphate (PIP2), which plays a critical role in regulating ion channels. This distinctive feature is further reinforced by the unique gating properties of Kir channels [12]. It is crucial to emphasize that the absence of PIP2 significantly affects various Kir channels, leading to a significant reduction in their activity. These findings suggest that PIP2 plays an essential role in maintaining normal activity of these channels [13]. Additionally, regulation of Kir channel pores involves pH reduction [14], protein kinase phosphorylation [1,15], and protein-protein interactions [16,17]. As a potential therapeutic target for various diseases, Kir channels have proven to be effective in the treatment of arrhythmias and hypertension through the use of certain medications that block the channels. Zacopride is a drug that activates Kir2.1 channels and shows promise in its potential as an antiarrhythmic agent [18]. However, inhibiting Kir channels with specific drugs like sulfonylureas and glinides has also been shown to improve insulin secretion in patients with type 2 diabetes.

## The significance of Kir channels in VSMCs and their expression and functional changes in the context of diseases

### Fundamental expression and traits of Kir channels in VSMCs

Numerous studies have demonstrated that Kir channels are prevalent in various cell types, ranging from excitatory to non-excitatory cells, and including VSMCs [19]. In the absence of external depolarization factors, such as pressure or vasoconstrictors, these channels remain open and help regulate Vm of arteries. The most relevant Kir channels to the vascular system are those from the Kir2.x (strong inward rectification) and Kir6.x (weak inward rectification) subgroups. In the cerebral and coronary arteries, Kir2.1 was found to be the most dominant channel expressed, while in the mesenteric artery, Kir2.1, Kir4.1, and Kir6.1 were found to be the most heavily expressed channels. According to reports, Ba^2+^ and Cs^+^ can specifically inhibit the Kir channel in SMCs, and the blocking effect is dependent on time and voltage [20]. Tennant et al [21] conducted a study on Kir channels in cultured human pulmonary artery smooth muscle cells (HPASMCs) and compared them to cloned Kir2.1 and Kir2.4 channels. They discovered that Kir current could be recorded in HPASMCs under physiological K^+^ gradient, and its current amplitude and reversal potential were sensitive to extracellular K^+^ concentration. The concentration of Ba^2+^ at 100 μM was found to have a significant impact on the inward current and Vm of HPASMCs, leading to a depolarization of approximately +10 mV. In the presence of 60 mM extracellular K^+^, Ba^2+^ was able to block the Kir current on HPASM cells, with a 50% inhibition concentration of 39.1 μM at a Vm of −100 mV, while for Kir2.1 and Kir2.4 clones, the 50% inhibition concentrations were noted as 3.9 μM and 65.6 μM at a Vm of −100 mV, respectively. Despite this, some K^+^ channel inhibitors such as 4-aminopyridine (4-AP) or tetraethylammonium (TEA) were found to have a negligible effect on Kir channels. It is pertinent to note that small resistance arteries in structures like the brain and coronary arteries experienced activation of Kir channels upon the increase of extracellular K^+^ concentration to 15 mM [22]. This increase in K^+^ concentration plays a crucial role in K^+^ mediated vasodilation. Targeted destruction of Kir2.1 and Kir2.2 genes has revealed the indispensability of Kir current in mediating K^+^ induced vasodilation. As such, studies have identified the Kir2.1 gene as necessary for Kir current and K^+^ induced cerebral artery dilation in arterial smooth muscle [23].

### The physiological functions of Kir channels in VSMCs

Arteries are comprised of two key types of cells, VSMCs and ECs, which work together in close coordination. Similar to cerebral arteries, both SMCs and ECs can detect specific blood flow patterns and work in tandem to regulate basal tension and cerebral blood flow. The establishment of arterial tension is intricately linked to Vm of the artery and the ion channels located on its membrane. Among these channels, Kir channels with robust inward rectification features are particularly critical [24].

The RP of VSMCs plays a crucial role in regulating cytoplasmic calcium levels and therefore the vascular tone. It is well established that there are variations in RP across different layers of the vasculature. In 2015, Yang et al [25] researched the underlying mechanism of RP heterogeneity in various vascular layers. They conducted a comparative analysis to examine the differences between the RPs of guinea pig spiral modiolar artery, brain arterioles, and mesenteric arteries, as well as the variations in the motor features of arteries. Their findings indicated that all three vessels exhibited strong Kir2.1 and Kir2.2 transcripts, as well as Kir2.1 immune markers. Nonetheless, the levels of Kir2.3 and Kir2.4 transcripts differed. They hypothesized that a subgroup of VSMCs in these vessels functionally express the Kir2.x channel, which forms the basis for the more negative RPs of ECs in these vessels. The varied bimodal RPs observed in these arterioles can be attributed to the heterogenous Kir channel function. The rapid regenerative exchange between the two RP states dependent on Kir channels may be a significant mechanism for the generation of vasomotor conduction/diffusion along the arteriolar axis. Lee et al [26] conducted a study on the regulatory effect of K^+^ channels on RP in gastric smooth muscle (HGCS). The results indicated that TEA and charybdotoxin had no impact on RP, indicating that the large conductance calcium-activated potassium channel (BKCa) did not play any role in regulating RP. The small conductance calcium-activated potassium channel (SKCa) selective blocker, Apamin, had no significant effect on membrane excitability. The Kv channel blocker, 4-AP, led to depolarization and increased the duration of the slow-wave potential. The study also found that Kv1.2 and Kv1.5 were present in human HGCS. The KATP blocker, Glibenclamide, did not induce depolarization, but the KATP opener, nicorandil, hyperpolarized HGCS, indicating that KATP was expressed but not fundamentally activated in HGCS. Notably, the study found that a low concentration of Kir blocker Ba^2+^ could induce significant depolarization of HGCS. Kir2.1 mRNA was expressed in HGCS, and Ba^2+^ sensitive current was expressed in the whole cell configuration. These findings suggest that the Kir channel is the only K^+^ channel that regulates RP in HGCS.

Fine-regulated processes are essential for maintaining cerebral blood flow, which depends on coordinated changes in arterial tone. In 2007, Wu et al [27] conducted a study on Kir channels, which are stimulated by depolarization and regulated by vasoactive stimuli. These stimuli are known to constrict intact cerebral arteries. Their preliminary experiments revealed that Ba^2+^ sensitive Kir currents in cerebral arterial VSMCs were activated within the physiological range of Vm. Inhibition of these currents resulted in arterial depolarization and contraction. Interestingly, depolarization and vasoconstrictive agonists did not have a significant effect on whole-cell Kir channel activity. However, hyposmotic challenge stimulation, which activated mechanically sensitive ion channels, led to the rapid and sustained inhibition of Kir currents. This finding suggests that mechanical stimulation plays a vital role in regulating the electrical and mechanical state of intact cerebral arteries by regulating protein kinase C (PKC) and Kir channel activity. Smooth muscle Vm plays a crucial role in this process, and Kir channels are believed to be a critical determiner. In order to better understand the role of Kir2.1 channels in cerebral artery tone development, Kowalewska et al [28] investigated the electrical and functional properties of vascular cells, blood vessels, and living tissues in tamoxifen-induced SMC-specific Kir2.1 knockout mice. The results indicated that the expression of Kir2.1 decreased significantly following knockout, while the expression of Kir2.2 was significantly more abundant. It was found that cerebral arterial myocytes exhibited a robust Ba^2+^ sensitive inward recirculating K^+^ current, which was observed regardless of whether Kir2.1 was present or knocked out. Interestingly, the myogenic response and K^+^ induced dilation of cerebral arteries remained uninhibited after knockout, leading to the conclusion that Kir2.2 played a more pivotal role in the functionality of SMCs. Studies have revealed that extracellular K^+^ acts as a speedy and efficient vasodilator. Recent research highlights the role of K^+^ release from astrocytic endfeet, which envelop the entire parenchymal vasculature, in the dynamic control of local cerebral blood flow (CBF) during neurovascular coupling (NVC). In 2015, Longden et al [29] put forward the hypothesis that vascular Kir channels are the primary extracellular K^+^ sensors that regulate CBF. They proposed that K^+^ is an ideal mediator of NVC, and Kir channels serve as effectors responsible for producing swift hyperpolarization and robust vasodilation of cerebral arterioles. Furthermore, they suggested that Kir channels, particularly those of the Kir 2 subtype, are located in both ECs and VSMCs of parenchymal arterioles. This dual positioning of Kir 2 channels enhances the potency of vasodilation in response to external K^+^. Recently, researchers [24,30] have made significant progress in understanding how cerebral arteries perceive hemodynamic changes. They have confirmed the prior findings [31–34] pertaining to the interaction between membrane lipids and Kir2.x channels that enable detection of alterations in the environment. Through their experiments, the team has demonstrated that the presence of PIP2 and cholesterol interacting with Kir2.x may respectively stabilize the channel in either the open or silent state. This interaction is critical in enabling specific channel populations to sense hemodynamic stimuli and trigger vasomotor responses, which regulate the basal perfusion forces in brain circulation.

Remarkably, Kir channels have also been identified in human bronchial SMCs [35], coronary arteries cells (CASMCs) [36], renal arterioles [37–39] and bladder small arteries [40]. These findings hold great potential for the advancement of novel therapies that focus on targeting Kir channels across different cell types. By doing so, it is believed that vascular tone function can be effectively enhanced, ultimately treating a range of cardiovascular diseases. In the study of the regulatory role of Kir, Park et al [36] investigated the effect of the vasoconstrictor angiotensin II (Ang II) on the whole-cell Kir current in SMCs isolated from small-diameter (<100 µm) CASMCs. They found that PKCα, not PKCβ, was expressed in small-diameter CASMCs, and Ang II inhibited the Kir channels by activating PKCα through the AT-1 receptor. The afferent and efferent arterioles regulate the inflow and outflow resistance of the glomerulus, acting in concert to control the glomerular capillary pressure and glomerular filtration rate. Research has revealed [37] that Kir2.1 mRNA and antibody markers are expressed by both types of renal vessels, along with inward Kir currents. Nonetheless, this current solely influences the reactivity of one type of arteriole to regulation by the other arteriole. The presence of Kir in output arterioles (a kind of resistance vessel that is not impacted by Vm) implies a novel function of this channel in the renal microcirculation. Chilton et al [38] investigated the role of Kir channels in the interlobular arteries (ILA) of the kidney. Their patch clamp experiments showed that myocytes in the incoming arterioles and distal ILAs exhibited similar large Kir currents, whereas these currents were absent in the proximal ILA myocytes. Kir current density was negatively correlated with the diameter of the ILA segment. They also confirmed that the myogenic response pattern of the ILA was consistent with the distribution of Kir currents (i.e. distal > intermediate > proximal), thus validating the increase in myogenic responsiveness of Kir channels with decreasing vessel diameter from the proximal to distal regions. Liu et al [39] studied the role of prostaglandins in regulating Kir activity in rat renal interlobar arteries (RIRAs). They measured intracellular Ca^2+^ concentration ([Ca^2+^]_i_) and Kir currents in freshly isolated RIRAs VSMCs, and the expression of Kir2.1 in RIRAs. They found that BaCl_2_ induced RIRAs contraction in a concentration-dependent manner and elevated [Ca^2+^]_i_ levels. In both resting and stimulated RIRAs, cyclooxygenase inhibition and thromboxane-prostaglandin receptor (TP) antagonism inhibited BaCl_2_-induced RIRA contraction, while nitric oxide synthase inhibition and de-endothelialization enhanced RIRA contraction. Tykocki et al [40] conducted a study on the role of Kir in regulating bladder vascular reactivity. They analyzed the myogenic activity of mouse bladder small arteries (BFAs) and the currents of cells isolated from them, and concluded that the activation of Kir channels could partially explain the dysregulation of hyperpolarization and associated BFAs. They suggested that the regulation of bladder vascular tone is independent of pressure, as pressure-induced depolarizing conductance cannot overcome the hyperpolarization mediated by Kir2.1. Additionally, researchers conducted a study to examine the functional activity of Kir channels and the impact of channel blockade on acetylcholine-induced vasodilation, a crucial measure of cellular communication, in the blood vessels supplying the hamster’s stretch muscle. Their findings indicate that smooth muscle Kir channels have a significant role in promoting intercellular communication in the resistance arteries of skeletal muscle [41].

### Compensatory changes of Kir channels in VSMCs during pathological conditions

Enhanced activity and expression of Kir channels in vascular tissues have been associated with various pathological conditions. Essential hypertension is often characterized by heightened vascular tone, which can be attributed to depolarization of VSMCs and shifts in the expression of ion channels that facilitate arterial constriction. Tajada et al [42] conducted a study that measured mRNA expression levels of Kir channels in various vascular beds of both phenotypically selected hypertensive (BPH) and normal blood pressure mice, and evaluated their current contribution to VSMCs excitability and mesenteric artery vascular tone. Their research suggested that reduced KATP current could be a significant factor in the remodeling of VSMCs in essential hypertension. Normal endothelium-dependent hyperpolarization (EDH) is mediated by SKCa and the intermediate conductance calcium-activated potassium channel (IKCa) within the endothelium that leads to vasodilation. In addition to electrical transmission through the smooth muscle-endothelial space junction, K^+^ released from the endothelium increases Kir conductivity of SMCs, which promotes EDH. Researchers [43] studied EDH-dependent relaxation of coronary arteries (CAS) and Kir currents in VSMCs in disease states like hypertension. Their findings confirmed a significant increase in Kir in CASMCs in SHR animals, suggesting that this compensation mechanism may partially offset the weaker relaxation of coronary vessels via endothelial SKCa and IKCa channels.

Functional adaptive enhancement of Kir channels has been observed in other cardiovascular disorders. A study by Park et al [44] in 2005 investigated the impact of acute hypoxia on Kir currents in rabbit coronary VSMCs and found that the currents were altered in response to acute hypoxia. It was observed that hypoxia triggered a rise in Kir currents in coronary VSMC through cyclic adenosine monophosphate (cAMP)- and protein kinase A (PKA)-dependent signaling cascades, which could assist in deciphering hypoxia-induced coronary vasodilation. It is reported that the amplitude of K^+^ current in mouse epigastric SMCs increased with age, reinforcing the integral role that Kir channels play in regulating RP and vasomotor tension. This increase in Kir current in older mice may serve to compensate for functional alterations in the abdominal wall superior epigastric arteries caused by aging. Tajada et al. also observed an upsurge in functional expression of Kir channels in the resistance artery smooth muscle cells of older mice skeletal muscle. This adaptation in response to aging may contribute to maintaining vasomotor tone and regulating blood flow during exercise. Spasticity in cerebral vessels has been reported in patients following subarachnoid hemorrhage (SAH). Weyer et al [45] investigated the mechanism of vasospasm, with a focus on the role of Kir channels. Their findings revealed an increase in both mRNA and protein expression of Kir 2.1 during vasospasm after SAH, which suggests that this increase is a functional adaptive response that aids in reducing vasospasm.

However, decreased expression and dysfunction of Kir channels in disease states have also been reported. In a study conducted by Bastide et al [46], Kir channels were observed in the cerebral VSMCs of spontaneous hypertensive stroke (SHRsp) rats and spontaneous hypertensive rats (SHR). The findings demonstrated that VSMCs obtained from SHRsp rats exhibited a significant and gradual reduction in Kir channel current density from 22 weeks of age, while the control rats maintained a stable Kir current density during the same period. This suggests that progressive damage to the Kir channels occurred in the cerebral VSMCs of SHRsp rats, leading to a decrease in Kir current density. Kim et al [47] discovered a decrease in Kir and Kv currents in coronary VSMCs of rats with pulmonary hypertension. The reduced Kir in septal branches of coronary SMCs indicates that, in the presence of heightened myocardial activity, the septal region becomes less effective in responding to vasodilation with moderate increases in extracellular K^+^ concentration. Sancho and his colleagues [48] observed limited expression of the Kir2.x channels in the human arteries of epileptic patients, which consequently has minimal impact on resting tone or the spread of vasomotor response. They propose that the absence of significant expression of Kir2.x may contribute to chronic cerebral stress in the epileptic cortex, thereby suggesting a close link between Kir channels and the pathogenesis of epilepsy. Longden et al [29] revealed the deleterious impact of stress on neurovascular coupling (NVC) and demonstrated that stress weakened the vasodilatory effect of arteriolar parenchymal vessels (PAs) on neuronal stimulation, while diminishing the vasodilation response of isolated PAs to extracellular K^+^. This was attributed to deficient smooth muscle Kir channel function in the ability to promote vasodilation, as evidenced by a decrease in Kir2.1 mRNA and smooth muscle Kir current density in PAs under stress conditions. The authors proposed that glucocorticoid signaling pathways induced by stress played a role in impeding cerebrovascular Kir channel opening and consequently destroyed neurovascular coupling.

**Figure 1.**
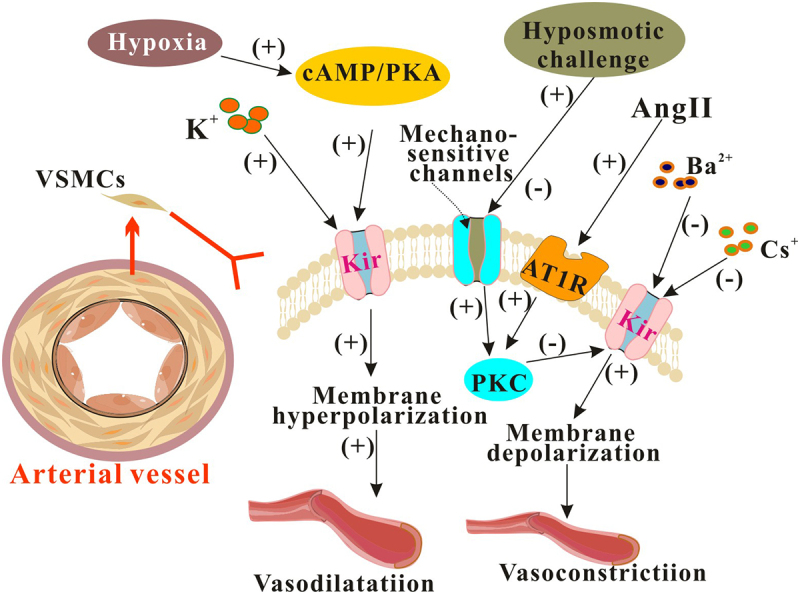
The Kir channels on SMCs regulates vasomotor activity. The Kir channels found on VSMCs are essential for regulating vasomotor activity, and their impact is even more pronounced in pathological conditions. Ang II: angiotensin II; AT1R: cAMP: cyclic adenosine monophosphate; PKA: Protein kinase A; PKC: Protein kinase C.

The main findings in functional expression of Kir channels in VSMCs and their changes under pathological influences were summarized in Figure 1.

## The significance of Kir channels in vascular ECs and their expression and functional changes in the context of diseases

### Fundamental expression and traits of Kir channels in vascular ECs

Studies have revealed that ECs exhibit a diverse range of K^+^ ion channels, including the Kir channels of the inward rectification family. These channels play a vital role in regulating vascular tone and controlling arterial blood pressure, and their electrophysiological properties, activation mechanisms and vascular wall expression are closely associated with these functions [49]. In 2003, Crane and his colleagues [50] conducted a study on the distribution of Kir channels in rat mesenteric arterioles using enzymatic isolation cells. Their findings indicated that Kir currents were exclusively present in the endothelial layer of rat mesenteric arterioles, with 30 μM BaCl_2_ effectively blocking Kir currents in these ECs. Conversely, no Kir currents were detected in VSMCs. An explanation for the apparent failure to detect current through Kir was that the channels were either very small or required agonist stimulation in order to be detected. The different K^+^ channel profiles reflect a real difference in the distribution of ion channels between SMCs and ECs of the mesenteric artery. In 2005, Fang et al [51] determined the composition of Kir2.x in human aortic endothelial cells (HAECs). They discovered that Kir2.1, Kir2.2, Kir2.3, and Kir2.4 were all expressed in HAECs. Additionally, it was found that Kir2.1 and Kir2.2 play a crucial role in determining the endogenous K^+^ conductance of HAECs while Kir2.2 is the primary conductance. Climent et al [52] investigated gap junction coupling in an integrated intact preparation and tested whether Kir channels modulate RP conductance in “*in situ*” ECs. The results suggest that the ECs layer of a large artery is electrically coupled, and Kir channels play an important role in RP in *in situ* ECs. In 2011, Jang et al [53] discovered Kir current in endothelial progenitor cells (EPCs) using whole-cell patch clamp technology. They found that the current was inhibited by 100 μM Ba^2+^ and 1 mM Cs^+^ and the inhibition resulted in endothelial cell depolarization. The study demonstrated that several subtypes of Kir channels, including Kir2.x, Kir3.x, Kir4.x, and Kir6.x, are present on endothelial cells. Furthermore, inhibition of Kir2.1 promoted the proliferation of EPCs. Multiple studies have indicated that capillary endothelial cells express Kir2.1 and Kir2.2, and may also express Kir2.3. William et al. have identified the presence of Kir2.1 protein on rat and mouse arteriole ECs, revealing it to be the fundamental Kir channel subunit expressed in both rat and mouse microvascular ECs [54]. Moreover, research has demonstrated that pulmonary artery endothelial cells (PAECs) primarily express Kir2.1 currents and that Kir2.1 channels are targeted by intracellular signaling mechanisms that are dependent on calmodulin (CaM)-dependent protein kinase II (CaMKII) [55].

### Physiological functions of Kir channels in vascular ECs

Research indicates that an increase in extracellular K^+^ concentration triggers the activation of Kir currents. These channels sense variations in extracellular K^+^ concentration, causing the membrane to become hyperpolarized and increasing the hyperpolarization created by other K^+^ channels and ion transporters. This amplification aids in regulating the Vm of ECs and other microvascular functions [54]. The preservation of the blood-brain barrier, formed by brain capillary endothelial cells (BCECs), is critical to ensuring proper brain homeostasis. Studies reveal that the activation of kir2.1 channels fosters the establishment of a deeply negative RP in BCECS-derived t-BBEC117 cells. This has been found to regulate the death of t-BBEC117 cells, indicating that an increase in Kir2.1 levels during pathological states can induce cell stress-induced death of BCECs cells [56]. Moreover, Fancher et al [57] discovered that fluid shear stress activated Kir2.1 channels in vascular ECs to induce downstream signal changes, leading to resistance artery vasodilation and serving a crucial role in atherosclerosis development. Furthermore, the expression of Kir2.1 channels in mesenteric artery ECs was found to mediate their response to external K^+^ ion concentration, impacting vasodilation. Inhibition or knockout of Kir channels can weaken endothelium-dependent vasodilation. To investigate the role of the Kir channel in the physiological response of ECs to blood flow, Ahn et al [6] examined the effects of the Kir2.1 channel on primary ECs from mouse mesenteric arteries. The study revealed that the Kir2.1 channel regulated vascular resistance and blood pressure, inhibited flow-induced vasodilation in human microvessels, and participated in mesenteric artery flow-induced vasodilation (FIV) in mice by affecting NO production. It has been discovered that in mice with endothelium-specific Kir2.1 knockout, mesenteric artery FIV was significantly decreased, indicating that Kir2.1 is a regulator of resistive artery FIV.

Endothelial cells are tightly electrically coupled to their neighboring cells by gap junctions allowing ion channel-induced changes in Vm to be conducted for considerable distances along the endothelial cell tube that lines arterioles and forms capillaries. According to research, Kir channels in mouse mesenteric arteries are also capable of transmitting electrical signals to superior arterioles through cell-cell conduction and can participate in vasodilation [54]. Numerous studies have highlighted the potential of Kir channels in ECs to enhance vascular dilatation induced by an increase in Ca^2+^ and activation of IKCa/SKCa channels, leading researchers to suggest that Kir channels could be targeted for the treatment of endothelial dysfunction [58]. Recent work by Jackson [54] summarized the expression of a range of ion channels by ECs at different sites within the microcirculation, with these ion channels playing a crucial role in ECs communication via heterocellular gap junctions. The electrical signals generated by ECs ion channels are transmitted to overlying mural cells, such as pericytes or VSMCs, where they can impact contractile activities. The process may involve the amplification of hyperpolarization induced by IKCa and SKCa activation via Kir channels.

### Compensatory changes of Kir channels in ECs during pathological conditions

Previous research has demonstrated that EDH induces hyperpolarization and vasodilation, and can spread throughout the endothelium in mesenteric arteries of rats. However, this response is weakened in SHR rats, and the same effect can be elicited through inhibition of Kir channels [59]. Although downregulation of Kv7.x has been observed in arterioles of hypertensive animals, its role in blood pressure regulation remains unclear. Moreover, alterations in the expression and activity of Kir and KATP channels within the vascular system of animal models with obesity have been shown to be associated with vasodilation [60]. Further studies have shown that aging altered cerebrovascular endothelial GPCR and K^+^ channel function, and that the effect of Kir channels on Vm in male endothelial cells was slightly lower than that in female cells. As individuals aged, the contribution of Kir channels in both sexes gradually declined, which was related to endothelial dysfunction [61].

The expression, function associated with impaired vasodilatation, and sensitivity of vascular K^+^ channel subtypes are impaired in individuals with obesity and metabolic syndrome, leading to dysfunction of both smooth muscle and endothelium [62]. It has been shown that endothelial dysfunction in hypercholesterolemia is associated with cholesterol-induced Kir2.1 inhibition [6]. This inhibition is believed to reduce flow-induced NO production and vasodilation, leading to further vascular complications. Interestingly, Kir2.1 has been found to have a protective role in the occurrence and development of atherosclerosis [63]. Ahn et al [62] tested EC Kir channel in normal and high fat fed mice, and discovered that down-regulation of Kir2.1 resulted in reduced vasodilation in the subcutaneous fat artery. Obesity has been shown to reduce the sensitivity of Kir channels in the ECs of visceral adipose arteries to shear force, and to significantly alter the structure of the glycocalyx. However, endothelial Kir2.1 differently in ECs of visceral and subcutaneous fatty arteries. Studies have revealed that in vivo hypercholesterolemia inhibits ECs Kir currents, and *in vitro*, high cholesterol lipoprotein inhibits Kir currents [64]. Alaaeddine et al [65] investigated the mechanism of endothelial injury in prediabetes and discovered that non-obese high-calorie-fed rat (HC-rat) models had decreased expression of Kir channels, increased ROS, and reduced eNOS activity. Aortic relaxation in HC-rat tissues, sensitive to NOS inhibition, was not affected by blocking Kir channels. In HC- rats with reduced serum cholesterol, Kir channel expression, endothelium-dependent relaxation, and BaCl_2_ sensitive components were improved, and ROS was decreased *in vitro*. They suggested that early metabolic challenge leads to reduced Kir-mediated endothelium-dependent hyperpolarization. Subsequently, Fancher et al [66] found that functional downregulation of endothelial Kir2.1 in mice decreased the response of mesenteric artery to blood flow, overexpression of Kir2.1 restored the blood flow response, and the sensitivity of Kir channel to blood flow in obese mice almost disappeared. The result indicated that the length of glycocalyx was also related to the activation of Kir channels, and the intraarterial glycocalyx became hard and thin in obese mice.

The Kir channel plays a crucial role in regulating neurovascular vasodilation. These channels are responsible for regulating the resting state of cells and the distance that electrical signals travel along arterial walls in the brain’s circulation. However, the expression of Kir2.x channels has been found to be limited in the cerebral arteries of epileptic patients, which can negatively impact the resting tone and vasomotor response diffusion [48]. In addition to epilepsy, Alzheimer’s disease (AD) and cerebrovascular disease (CVD) also affect Kir channel function in ECs. Without substantive expression, Kir2.x channels were unable to govern arterial tone or conduction. The damage is attributed to oxidative stress and inflammation. The results of vascular endothelial Kir channel-mediated K^+^ induced arterial dilation showed that oxidative stress and inflammation impaired the flow-activation of Kir, particularly the Kir2.1 channel. This impairment reduced vasodilation response, while inflammation induced overexpression of Kir2.1 in mice [67]. In their research, Hakim and colleagues discovered a decrease in the G-protein-coupled receptors and Ca^2+^ activated potassium channels (SKCa and IKCa) and Kir2.x channels during progressive AD pathology. Using newly isolated ECs of rat posterior cerebral artery, they determined that the function of SKCa/IKCa channels in male cerebral vascular ECs was enhanced by about 20% during the pathological process of AD, while that in females remained stable. Vascular Kir channels, which regulate cerebral blood flow and perfusion, were found to be interdependent with SKCa channel activation. However, the contribution of the Kir channel to Vm in AD decreased by about 50% compared to young age, which may be due to changes in membrane lipid and cholesterol content in the development of AD [68]. Recent studies have reported on the impact of Kir channel activity on ECs coupling before and during AD. The findings indicate that Kir channel activity in brain ECs can regulate the bidirectional propagation of vascular response signals during AD pathology, thereby enhancing the regulation of ECs coupling [69].

**Figure 2.**
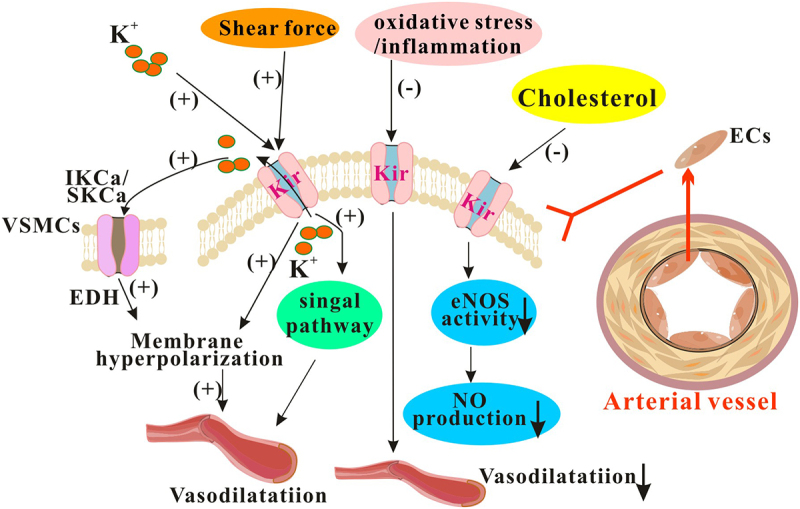
The Kir channels on vascular ECs regulates vasomotor activity. The Kir channels found in vascular ECs are essential for maintaining proper vasomotor activity. However, their function can be compromised under pathological conditions. EDH: endothelium-dependent vasodilation; IKCa: Intermediate conductance calcium-activated potassium channel; SKCa: Small conductance calcium-activated potassium channel.

The main findings in functional expression of Kir channels in vascular ECs and their changes under pathological influences were summarized in Figure 2.

## Kir channels and stem cells

Stem cells possess the remarkable ability to continuously grow and renew themselves, and are capable of developing into diverse types of mature tissue cells. Within the blood vessel wall, vascular resident stem cells exist within all three layers, contributing to the formation of blood vessels in normal physiological scenarios, while also playing a role in remodeling them during pathological conditions. The impact of some ion channels on stem cell function has been previously documented [70]. Although the existence of Kir channels on stem cells has not been extensively studied, there is some evidence to suggest their presence.

In 2011, Jang et al [53] published a study on the functional expression of Kir channels in endothelial progenitor cells (EPCs). The researchers discovered that Kir currents in EPCs were inhibited by Kir blockers, in a dose-dependent manner. Specifically, at a Vm of −140 mV, Ba^2+^ (100 μM) inhibited 91.2 ± 1.4% of the current, while Cs^+^ (1 mM) inhibited 76.1 ± 6.9% of the current. Furthermore, using DiBAC(3) – a fluorescent indicator of Vm – the researchers demonstrated that Ba^2+^ induced fluorescence enhancement of EPCs (at 10 μM, 123 ± 2.8%), which suggested depolarization of EPCs. In conducting functional experiments, they discovered that the presence of Ba^2+^ resulted in a dose-dependent decrease in the number of tubes formed by EPCs on Matrigel. Additionally, the proliferation of EPCs showed a corresponding dose-dependent increase, with specific inhibition of Kir2.1 achieved by small interfering RNA yielding further promotion of EPCs proliferation. These findings strongly suggest that multiple types of Kir may be expressed in EPCs, enabling them to regulate endothelial function and proliferation of ECs effectively. It has been reported that controlling the Kir channel of EPCs can regulate their endothelial function and proliferation. When Kir2.1 channels are inhibited, EPCs undergo depolarization, which induces autophagy and promotes differentiation into ECs. Conversely, the activation of Kir2.1 channels leads to EPCs hyperpolarization. In addition, the Akt/mTOR/Snail signaling pathway is activated to induce EPC transdifferentiation into mesenchymal cells, specifically pericytes, which can regulate and maintain stemness [71]. Researchers have discovered that slightly increasing the expression of Kir2.1 leads to the promotion of stem cell cytokines ZFX and NS, as well as the inhibition of age-related β-galactosidase. Additionally, the study revealed that the upregulation of Kir2.1 also results in an increase in pericellular markers, such as NG2, PDGFRβ and Desmin. Using adenoviral-mediated overexpression, the researchers found that Kir2.1 enhances the norepinephrine contractile response of EPCs. Furthermore, the study suggests a crucial connection between the Kir2.1 channel’s activity and EPCs’ transdifferentiation into mesenchymal cells, specifically pericytes. This process is further facilitated through the activation of the Akt/mTOR/Snail signaling pathway, promoting EPCs’ endothelial to-mesenchymal transition (EndoMT). The Kir channel-specific inhibitor ML133 was employed to inhibit Kir2.1, and the results showed that inhibition or knockdown of Kir2.1 led to changes in Vm, which in turn promoted adhesion and migration of rat bone marrow-derived EPCs (BMEPCs). Furthermore, ML133 or Kir2.1shRNA treatment enhanced NO production and capillary formation and facilitated BMEPC differentiation into ECs. Following transplantation in ML133 pretreated EPCs or Kir2.1 knockout EPCs, there was a decrease in neointima formation post-arterial injury [72].

Although there is limited research on Kir in vascular stem cells, studies on other types of stem cells have provided insights into the potential roles and mechanisms of Kir in stem cell biology. In fact, bone marrow mesenchymal stem cells (MSCs) are believed to offer great potential as a cell source for cardiomyoplasty. According to research by Tao et al [73], who utilized patch clamp technique, RT-PCR and western blotting, undifferentiated MSCs from mice were found to possess three types of ion channels: KCa, Kir and chlorine ion channels (Cl^−^). This team identified corresponding ion channel genes and proteins in these cells, which were KCa3.1 for KCa, Kir2.1 for Kir and Clcn3 for Cl. The study sought to assess the impact of long-term in vitro culture on ion channels of human MSCs (hBM-MSCs). Specifically, the experiment involved culturing cells from eight patients with amyotrophic lateral sclerosis (ALS) for ten generations. Although all hBM-MSCs had multiple MSC markers, differences in the distribution of functional ion channels were observed across cells. Notably, four types of K^+^ currents were identified in the cells- noisy Ca^2+^ activated K^+^ current, transient outward K^+^ current, delayed rectifying K^+^ current, and Kir current. Besides, TTX-sensitive Na+ current was recorded during the experiments. Hinard et al [74] have reported that the earliest detectable event leading to human myoblast differentiation is the up-regulation of Kir2.1 channel activity, which causes a negative shift, or hyperpolarization, of RP of myoblasts. They have found that while the Kir2.1 channel is already present on the cell membrane of proliferating, undifferentiated human myoblasts, it remains silent until dephosphorylation of tyrosine site 242 on the Kir2.1 channel protein triggers differentiation. Andersen’s syndrome is a rare disorder that affects muscles, bones, and the heart, and it is caused by mutations that result in the loss of function of Kir2.1. Scientists have utilized induced pluripotent stem cells from Andersen’s syndrome to investigate the cellular and molecular events that occur during osteoblastic and chondrogenic differentiation. The research has revealed that the loss of the Kir2.1 channel disrupts the bone morphogenetic protein signaling pathway. However, there is hope, as the study also demonstrated that restoring the Kir2.1 channel in Andersen’s syndrome cells can reverse the negative effects and restore normal bone and cartilage behavior by re-regulating major gene expressions.

## Summary

Bioelectrical signals play a crucial role in determining cellular behavior, with ion channels serving as the foundation of these signals. While there is some understanding of the relationship between functional vascular ion channel expression and vascular behavior, further exploration is needed. Kir channels, a type of ion channel responsible for maintaining the resting potential and signal transduction in most cells, are directly related to normal physiological functions of blood vessels and the development of certain diseases. Without the involvement of Kir channels, the ability of Kir to promote vasodilation in the circulatory system would be difficult to achieve. Different subtypes of Kir channels on VSMCs and ECs regulate vasomotor activity and blood flow supply. Advancements in understanding Kir channels are being made through various approaches such as analyzing their structure, studying their electrical activity using electrophysiological techniques, investigating their function using molecular biology techniques, and understanding their role in disease through the examination of specifically expressed subtypes in vascular tissues using transgenic animals. These findings have great potential to shed light on new treatment options for ion channel diseases and identify new targets for drug therapies.

## Data Availability

The authors confirm that the data supporting the findings of this study are available within the article.

## References

[1] Hibino H, Inanobe A, Furutani K, et al. Inwardly rectifying potassium channels: their structure, function, and physiological roles. Physiol Rev. 2010;90(1):291–16. doi: 10.1152/physrev.00021.2009 20086079

[2] Walsh KB. Screening Technologies for Inward Rectifier Potassium Channels: Discovery of New Blockers and Activators. SLAS Discov. 2020;25(5):420–433. doi: 10.1177/2472555220905558 32292089

[3] Meng XY, Liu S, Cui M, et al. The molecular mechanism of opening the Helix Bundle Crossing (HBC) gate of a Kir channel. Sci Rep. 2016;6(1):29399. doi: 10.1038/srep29399 27439597 PMC4954981

[4] Rapedius M, Fowler PW, Shang L, et al. H bonding at the helix-bundle crossing controls gating in Kir potassium channels. Neuron. 2007;55(4):602–614. doi: 10.1016/j.neuron.2007.07.026 17698013 PMC1950231

[5] Zuo D, Chen K, Zhou M, et al. Kir2.1 and K2P1 channels reconstitute two levels of resting membrane potential in cardiomyocytes. J Physiol. 2017;595(15):5129–5142. doi: 10.1113/JP274268 28543529 PMC5538241

[6] Ahn SJ, Fancher IS, Granados ST, et al. Cholesterol-induced suppression of endothelial Kir channels is a driver of impairment of arteriolar flow-induced vasodilation in humans. Hypertension. 2022;79(1):126–138. doi: 10.1161/HYPERTENSIONAHA.121.17672 34784737 PMC8845492

[7] Akyuz E, Koklu B, Uner A, et al. Envisioning the role of inwardly rectifying potassium (Kir) channel in epilepsy. J Neurosci Res. 2022;100(2):413–443. doi: 10.1002/jnr.24985 34713909

[8] Leem YE, Jeong HJ, Kim HJ, et al. Cdo regulates surface expression of Kir2.1 K+ channel in myoblast differentiation. PLoS One. 2016;11(7):e0158707. doi: 10.1371/journal.pone.0158707 27380411 PMC4933383

[9] Hebert SC, Desir G, Giebisch G, et al. Molecular diversity and regulation of renal potassium channels. Physiol Rev. 2005;85(1):319–371. doi: 10.1152/physrev.00051.2003 15618483 PMC2838721

[10] Marmolejo-Murillo LG, Arechiga-Figueroa IA, Moreno-Galindo EG, et al. Kir4.1/Kir5.1 channels possess strong intrinsic inward rectification determined by a voltage-dependent K±flux gating mechanism. J Gen Physiol. 2021;153(5). doi: 10.1085/jgp.201912540 PMC8025212 33822868

[11] Noujaim SF, Stuckey JA, Ponce-Balbuena D, et al. Specific residues of the cytoplasmic domains of cardiac inward rectifier potassium channels are effective antifibrillatory targets. FASEB J. 2010;24(11):4302–4312. doi: 10.1096/fj.10-163246 20585026 PMC2974416

[12] Ha J, Xu Y, Kawano T, et al. Hydrogen sulfide inhibits Kir2 and Kir3 channels by decreasing sensitivity to the phospholipid phosphatidylinositol 4,5-bisphosphate (PIP(2)). J Biol Chem. 2018;293(10):3546–3561. doi: 10.1074/jbc.RA117.001679 29317494 PMC5846148

[13] Suh BC, Hille B. PIP2 is a necessary cofactor for ion channel function: how and why? Annu Rev Biophys. 2008;37(1):175–195. doi: 10.1146/annurev.biophys.37.032807.125859 18573078 PMC2692585

[14] Dahlmann A, Li M, Gao Z, et al. Regulation of Kir channels by intracellular pH and extracellular K(+): mechanisms of coupling. J Gen Physiol. 2004;123(4):441–454. doi: 10.1085/jgp.200308989 15051808 PMC2217465

[15] Gada KD, Logothetis DE. PKC regulation of ion channels: The involvement of PIP(2). J Biol Chem. 2022;298(6):102035. doi: 10.1016/j.jbc.2022.102035 35588786 PMC9198471

[16] Tucker SJ, Ashcroft FM. Mapping of the physical interaction between the intracellular domains of an inwardly rectifying potassium channel, Kir6.2. J Biol Chem. 1999;274(47):33393–33397. doi: 10.1074/jbc.274.47.33393 10559219

[17] Dascal N, Kahanovitch U. The roles of gbetagamma and galpha in gating and regulation of GIRK channels. Int Rev Neurobiol. 2015;123:27–85. 26422982 10.1016/bs.irn.2015.06.001

[18] Lin Y, Li J, Zhu B, et al. Zacopride exerts an antiarrhythmic effect by specifically stimulating the cardiac inward rectifier potassium current in rabbits: exploration of a new antiarrhythmic strategy. Curr Pharm Des. 2020;26(44):5746–5754. doi: 10.2174/1381612826666200701135508 32611299

[19] Dogan MF, Yildiz O, Arslan SO, et al. Potassium channels in vascular smooth muscle: a pathophysiological and pharmacological perspective. Fundam Clin Pharmacol. 2019;33(5):504–523. doi: 10.1111/fcp.12461 30851197

[20] Cao C, Goo JH, Lee-Kwon W, et al. Vasa recta pericytes express a strong inward rectifier K+ conductance. Am J Physiol Regul Integr Comp Physiol. 2006;290(6):R1601–1607. doi: 10.1152/ajpregu.00877.2005 16439665

[21] Tennant BP, Cui Y, Tinker A, et al. Functional expression of inward rectifier potassium channels in cultured human pulmonary smooth muscle cells: evidence for a major role of Kir2.4 subunits. J Membr Biol. 2006;213(1):19–29. doi: 10.1007/s00232-006-0037-y 17347781 PMC1973150

[22] Gonzalez C, Baez-Nieto D, Valencia I, et al. K(+) channels: function-structural overview. Compr Physiol. 2012;2(3):2087–2149. 23723034 10.1002/cphy.c110047

[23] Zaritsky JJ, Eckman DM, Wellman GC, et al. Targeted disruption of Kir2.1 and Kir2.2 genes reveals the essential role of the inwardly rectifying K(+) current in K(+)-mediated vasodilation. Circ Res. 2000;87(2):160–166. doi: 10.1161/01.RES.87.2.160 10904001

[24] Sancho M, Fletcher J, Welsh DG. Inward rectifier potassium channels: membrane lipid-dependent mechanosensitive gates in brain vascular cells. Front Cardiovasc Med. 2022;9:869481. doi: 10.3389/fcvm.2022.869481 35419431 PMC8995785

[25] Yang Y, Chen F, Karasawa T, et al. Diverse Kir expression contributes to distinct bimodal distribution of resting potentials and vasotone responses of arterioles. PLoS One. 2015;10(5):e0125266. doi: 10.1371/journal.pone.0125266 25938437 PMC4418701

[26] Lee JY, Ko EJ, Ahn KD, et al. The role of K(+) conductances in regulating membrane excitability in human gastric corpus smooth muscle. Am J Physiol Gastrointest Liver Physiol. 2015;308(7):G625–633. doi: 10.1152/ajpgi.00220.2014 25591864 PMC4385896

[27] Wu BN, Luykenaar KD, Brayden JE, et al. Hyposmotic challenge inhibits inward rectifying K+ channels in cerebral arterial smooth muscle cells. Am J Physiol Heart Circ Physiol. 2007;292(2):H1085–1094. doi: 10.1152/ajpheart.00926.2006 17056667

[28] Kowalewska PM, Fletcher J, Jackson WF, et al. Genetic ablation of smooth muscle KIR2.1 is inconsequential to the function of mouse cerebral arteries. J Cereb Blood Flow Metab. 2022;42(9):1693–1706. doi: 10.1177/0271678X221093432 35410518 PMC9441723

[29] Longden TA, Nelson MT. Vascular inward rectifier K+ channels as external K+ sensors in the control of cerebral blood flow. Microcirculation. 2015;22(3):183–196. doi: 10.1111/micc.12190 25641345 PMC4404517

[30] Sancho M, Fabris S, Hald BO, et al. Membrane lipid-KIR2.X channel interactions enable hemodynamic sensing in cerebral arteries. Arterioscler Thromb Vasc Biol. 2019;39(6):1072–1087. doi: 10.1161/ATVBAHA.119.312493 31043073

[31] D’Avanzo N, Hyrc K, Enkvetchakul D, et al. Enantioselective protein-sterol interactions mediate regulation of both prokaryotic and eukaryotic inward rectifier K+ channels by cholesterol. PLoS One. 2011;6(4):e19393. doi: 10.1371/journal.pone.0019393 21559361 PMC3084843

[32] D’Avanzo N, Cheng WW, Doyle DA, et al. Direct and specific activation of human inward rectifier K+ channels by membrane phosphatidylinositol 4,5-bisphosphate. J Biol Chem. 2010;285(48):37129–37132. doi: 10.1074/jbc.C110.186692 20921230 PMC2988318

[33] Romanenko VG, Fang Y, Byfield F, et al. Cholesterol sensitivity and lipid raft targeting of Kir2.1 channels. Biophys J. 2004;87(6):3850–3861. doi: 10.1529/biophysj.104.043273 15465867 PMC1304896

[34] Shyng SL, Nichols CG. Membrane phospholipid control of nucleotide sensitivity of KATP channels. Science. 1998;282(5391):1138–1141. doi: 10.1126/science.282.5391.1138 9804554

[35] Oonuma H, Iwasawa K, Iida H, et al. Inward rectifier K(+) current in human bronchial smooth muscle cells: inhibition with antisense oligonucleotides targeted to Kir2.1 mRNA. Am J Respir Cell Mol Biol. 2002;26(3):371–379. doi: 10.1165/ajrcmb.26.3.4542 11867346

[36] Park WS, Kim N, Youm JB, et al. Angiotensin II inhibits inward rectifier K+ channels in rabbit coronary arterial smooth muscle cells through protein kinase Calpha. Biochem Biophys Res Commun. 2006;341(3):728–735. doi: 10.1016/j.bbrc.2006.01.026 16442501

[37] Chilton L, Loutzenhiser K, Morales E, et al. Inward rectifier K(+) currents and Kir2.1 expression in renal afferent and efferent arterioles. J Am Soc Nephrol. 2008;19(1):69–76. doi: 10.1681/ASN.2007010039 18178799 PMC2391029

[38] Chilton L, Smirnov SV, Loutzenhiser K, et al. Segment-specific differences in the inward rectifier K(+) current along the renal interlobular artery. Cardiovasc Res. 2011;92(1):169–177. doi: 10.1093/cvr/cvr179 21697146

[39] Liu Y, Wang Y, Guo P, et al. Prostanoids contribute to regulation of inwardly rectifying K(+) channels in intrarenal arterial smooth muscle cells. Life Sci. 2020;250:117586. doi: 10.1016/j.lfs.2020.117586 32222464

[40] Tykocki NR, Bonev AD, Longden TA, et al. Inhibition of vascular smooth muscle inward-rectifier K(+) channels restores myogenic tone in mouse urinary bladder arterioles. Am J Physiol Renal Physiol. 2017;312(5):F836–F847. doi: 10.1152/ajprenal.00682.2016 28148533 PMC5451557

[41] Jantzi MC, Brett SE, Jackson WF, et al. Inward rectifying potassium channels facilitate cell-to-cell communication in hamster retractor muscle feed arteries. Am J Physiol Heart Circ Physiol. 2006;291(3):H1319–1328. doi: 10.1152/ajpheart.00217.2006 16617135

[42] Tajada S, Cidad P, Moreno-Dominguez A, et al. High blood pressure associates with the remodelling of inward rectifier K+ channels in mice mesenteric vascular smooth muscle cells. J Physiol. 2012;590(23):6075–6091. doi: 10.1113/jphysiol.2012.236190 22966162 PMC3530118

[43] Kim HJ, Yin MZ, Cho S, et al. Increased inward rectifier K(+) current of coronary artery smooth muscle cells in spontaneously hypertensive rats; partial compensation of the attenuated endothelium-dependent relaxation via Ca(2+) -activated K(+) channels. Clin Exp Pharmacol Physiol. 2020;47(1):38–48. doi: 10.1111/1440-1681.13168 31444788

[44] Park WS, Han J, Kim N, et al. Activation of inward rectifier K+ channels by hypoxia in rabbit coronary arterial smooth muscle cells. Am J Physiol Heart Circ Physiol. 2005;289(6):H2461–2467. doi: 10.1152/ajpheart.00331.2005 16284107

[45] Weyer GW, Jahromi BS, Aihara Y, et al. Expression and function of inwardly rectifying potassium channels after experimental subarachnoid hemorrhage. J Cereb Blood Flow Metab. 2006;26(3):382–391. doi: 10.1038/sj.jcbfm.9600193 16079788

[46] Bastide M, Ouk T, Petrault O, et al. Time-induced progressive alteration of kir current in cerebral smooth muscle cells of stroke-prone spontaneously hypertensive rats. Int J Hypertens. 2013;2013:849750. doi: 10.1155/2013/849750 23710341 PMC3655577

[47] Kim SE, Yin MZ, Kim HJ, et al. Decreased inward rectifier and voltage-gated K(+) currents of the right septal coronary artery smooth muscle cells in pulmonary arterial hypertensive rats. Korean J Physiol Pharmacol. 2020;24(1):111–119. doi: 10.4196/kjpp.2020.24.1.111 31908580 PMC6940494

[48] Sancho M, Gao Y, Hald BO, et al. An assessment of KIR channel function in human cerebral arteries. Am J Physiol Heart Circ Physiol. 2019;316(4):H794–H800. doi: 10.1152/ajpheart.00022.2019 30681365

[49] Kohler R, Ruth P. Endothelial dysfunction and blood pressure alterations in K±channel transgenic mice. Pflugers Arch. 2010;459(6):969–976. doi: 10.1007/s00424-010-0819-z 20349244

[50] Crane GJ, Walker SD, Dora KA, et al. Evidence for a differential cellular distribution of inward rectifier K channels in the rat isolated mesenteric artery. J Vasc Res. 2003;40(2):159–168. doi: 10.1159/000070713 12808352

[51] Fang Y, Schram G, Romanenko VG, et al. Functional expression of Kir2.X in human aortic endothelial cells: the dominant role of Kir2.2. Am J Physiol Cell Physiol. 2005;289(5):C1134–1144. doi: 10.1152/ajpcell.00077.2005 15958527

[52] Climent B, Zsiros E, Stankevicius E, et al. Intact rat superior mesenteric artery endothelium is an electrical syncytium and expresses strong inward rectifier K+ conductance. Biochem Biophys Res Commun. 2011;410(3):501–507. doi: 10.1016/j.bbrc.2011.06.011 21679686

[53] Jang SS, Park J, Hur SW, et al. Endothelial progenitor cells functionally express inward rectifier potassium channels. Am J Physiol Cell Physiol. 2011;301(1):C150–161. doi: 10.1152/ajpcell.00002.2010 21411724

[54] Jackson WF. Endothelial ion channels and cell-cell communication in the microcirculation. Front Physiol. 2022;13:805149. doi: 10.3389/fphys.2022.805149 35211031 PMC8861442

[55] Qu L, Yu L, Wang Y, et al. Inward Rectifier K+ currents are regulated by CaMKII in endothelial cells of primarily cultured bovine pulmonary arteries. PLoS One. 2015;10(12):e0145508. doi: 10.1371/journal.pone.0145508 26700160 PMC4689359

[56] Kito H, Yamazaki D, Ohya S, et al. Up-regulation of K(ir)2.1 by ER stress facilitates cell death of brain capillary endothelial cells. Biochem Biophys Res Commun. 2011;411(2):293–298. doi: 10.1016/j.bbrc.2011.06.128 21726538

[57] Fancher IS, Levitan I. Endothelial inwardly-rectifying K(+) channels as a key component of shear stress-induced mechanotransduction. Curr Top Membr. 2020;85:59–88. 32402645 10.1016/bs.ctm.2020.02.002

[58] Sonkusare SK, Dalsgaard T, Bonev AD, et al. Inward rectifier potassium (Kir2.1) channels as end-stage boosters of endothelium-dependent vasodilators. J Physiol. 2016;594(12):3271–3285. doi: 10.1113/JP271652 26840527 PMC4908010

[59] Goto K, Rummery NM, Grayson TH, et al. Attenuation of conducted vasodilatation in rat mesenteric arteries during hypertension: role of inwardly rectifying potassium channels. J Physiol. 2004;561(Pt 1):215–231. doi: 10.1113/jphysiol.2004.070458 15550469 PMC1665331

[60] Climent B, Simonsen U, Rivera L. Effects of obesity on vascular potassium channels. Curr Vasc Pharmacol. 2014;12(3):438–452. doi: 10.2174/1570161112666140423221622 24846233

[61] Hakim MA, Chum PP, Buchholz JN, et al. Aging alters cerebrovascular endothelial GPCR and K+ channel function: divergent role of biological sex. J Gerontol A Biol Sci Med Sci. 2020;75(11):2064–2073. doi: 10.1093/gerona/glz275 31760422 PMC7566512

[62] Ahn SJ, Le Master E, Lee JC, et al. Differential effects of obesity on visceral versus subcutaneous adipose arteries: role of shear-activated Kir2.1 and alterations to the glycocalyx. Am J Physiol Heart Circ Physiol. 2022;322(2):H156–H166. doi: 10.1152/ajpheart.00399.2021 34890278 PMC8742723

[63] Fancher IS, Ahn SJ, Adamos C, et al. Hypercholesterolemia-induced loss of flow-induced vasodilation and lesion formation in apolipoprotein E-Deficient mice critically depend on inwardly rectifying K(+) channels. J Am Heart Assoc. 2018;7(5). doi: 10.1161/JAHA.117.007430 PMC5866319 29502106

[64] Fang Y, Mohler ER 3rd, Hsieh E, et al. Hypercholesterolemia suppresses inwardly rectifying K+ channels in aortic endothelium in vitro and in vivo. Circ Res. 2006;98(8):1064–1071. doi: 10.1161/01.RES.0000218776.87842.43 16556870

[65] Alaaeddine R, Elkhatib MAW, Mroueh A, et al. Impaired Endothelium-dependent hyperpolarization underlies endothelial dysfunction during early metabolic challenge: increased ROS generation and possible interference with NO function. J Pharmacol Exp Ther. 2019;371(3):567–582. doi: 10.1124/jpet.119.262048 31511364

[66] Fancher IS, Le Master E, Ahn SJ, et al. Impairment of flow-sensitive inwardly rectifying K(+) channels via disruption of glycocalyx mediates obesity-induced endothelial dysfunction. Arterioscler Thromb Vasc Biol. 2020;40(9):e240–e255. doi: 10.1161/ATVBAHA.120.314935 32698687 PMC7503211

[67] Lacalle-Aurioles M, Trigiani LJ, Bourourou M, et al. Alzheimer’s disease and cerebrovascular pathology alter inward rectifier potassium (K(IR) 2.1) channels in endothelium of mouse cerebral arteries. Br J Pharmacol. 2022;179(10):2259–2274. doi: 10.1111/bph.15751 34820829 PMC9304142

[68] Hakim MA, Behringer EJ, Aguayo L. Development of Alzheimer’s disease progressively alters sex-dependent KCa and sex-independent KIR channel function in cerebrovascular endothelium. J Alzheimers Dis. 2020;76(4):1423–1442. doi: 10.3233/JAD-200085 32651315 PMC7709862

[69] Hakim MA, Behringer EJ. K(IR) channel regulation of electrical conduction along cerebrovascular endothelium: Enhanced modulation during Alzheimer’s disease. Microcirculation. 2023;30(1):e12797. doi: 10.1111/micc.12797 36577656 PMC9885900

[70] Zhang M, Che C, Cheng J, et al. Ion channels in stem cells and their roles in stem cell biology and vascular diseases. J Mol Cell Cardiol. 2022;166:63–73. doi: 10.1016/j.yjmcc.2022.02.002 35143836

[71] Cui X, Li X, He Y, et al. Slight up-regulation of Kir2.1 channel promotes endothelial progenitor cells to transdifferentiate into a pericyte phenotype by Akt/mTOR/Snail pathway. J Cell Mol Med. 2021;25(21):10088–10100. doi: 10.1111/jcmm.16944 34592781 PMC8572793

[72] Zhang X, Cui X, Li X, et al. Inhibition of Kir2.1 channel-induced depolarization promotes cell biological activity and differentiation by modulating autophagy in late endothelial progenitor cells. J Mol Cell Cardiol. 2019;127:57–66. doi: 10.1016/j.yjmcc.2018.11.005 30447228

[73] Tao R, Lau CP, Tse HF, et al. Functional ion channels in mouse bone marrow mesenchymal stem cells. Am J Physiol Cell Physiol. 2007;293(5):C1561–1567. doi: 10.1152/ajpcell.00240.2007 17699636

[74] Hinard V, Belin D, Konig S, et al. Initiation of human myoblast differentiation via dephosphorylation of Kir2.1 K+ channels at tyrosine 242. Development. 2008;135(5):859–867. doi: 10.1242/dev.011387 18216177

